# Cellulose Nanofiber-Based Hydrogels Embedding 5-FU Promote Pyroptosis Activation in Breast Cancer Cells and Support Human Adipose-Derived Stem Cell Proliferation, Opening New Perspectives for Breast Tissue Engineering

**DOI:** 10.3390/pharmaceutics13081189

**Published:** 2021-08-01

**Authors:** Liliana-Roxana Balahura, Sorina Dinescu, Mihaela Balaș, Alexandra Cernencu, Adriana Lungu, George Mihail Vlăsceanu, Horia Iovu, Marieta Costache

**Affiliations:** 1 Department of Biochemistry and Molecular Biology, University of Bucharest, 050095 Bucharest, Romania; roxana.balahura@bio.unibuc.ro (L.-R.B.); mihaela.balas@bio.unibuc.ro (M.B.); marieta.costache@bio.unibuc.ro (M.C.); 2 Department of Immunology, National Institute for Research and Development in Biomedical Pathology and Biomedical Sciences “Victor Babes”, 050096 Bucharest, Romania; 3 Research Institute of University of Bucharest, 050107 Bucharest, Romania; 4 Advanced Polymer Materials Group, University Politehnica of Bucharest, 011061 Bucharest, Romania; alex.cernencu@gmail.com (A.C.); adriana.lungu@upb.ro (A.L.); vlasceanu.georgemihail@yahoo.ro (G.M.V.); horia.iovu@upb.ro (H.I.)

**Keywords:** 5-fluorouracil, breast cancer, inflammasome, caspase-1, p53, tissue regeneration, cellulose nanofibers, pectin, 3D scaffolds

## Abstract

The structure and biocompatibility analysis of a hydrogel based on cellulose nanofibers (CNFs) combined with alginate/pectin (A.CNF or P.CNF) and enriched with 1% or 5% 5-FU revealed more favorable properties for the cellular component when pectin was dispersed within CNFs. 5-Fluorouracil (5-FU) is an antimetabolite fluoropyrimidine used as antineoplastic drug for the treatment of multiple solid tumors. 5-FU activity leads to caspase-1 activation, secretion and maturation of interleukins (IL)-1, IL-18 and reactive oxygen species (ROS) generation. Furthermore, the effects of embedding 5-FU in P.CNF were explored in order to suppress breast tumor cell growth and induce inflammasome complex activation together with extra- and intracellular ROS generation. Exposure of tumor cells to P.CNF/5-FU resulted in a strong cytotoxic effect, an increased level of caspase-1 released in the culture media and ROS production—the latter directly proportional to the concentration of anti-tumor agent embedded in the scaffolds. Simultaneously, 5-FU determined the increase of p53 and caspase-1 expressions, both at gene and protein levels. In conclusion, P.CNF/5-FU scaffolds proved to be efficient against breast tumor cells growth due to pyroptosis induction. Furthermore, biocompatibility and the potential to support human adipose-derived stem cell growth were demonstrated, suggesting that these 3D systems could be used in soft tissue reconstruction post-mastectomy.

## 1. Introduction

Breast cancer (BC) represents the most frequent cause of death among women. It is also a heterogeneous disease with a high cell proliferation rate [1]. In the medical field, the assessment of breast tumor markers is the protocol most frequently used to establish diagnosis and prognosis. Some specific molecular markers—such as p53, Breast Cancer Gene 1 and 2 (BRCA1, BRCA2), nuclear factor kappa-light-chain-enhancer of activated B cells (NF-kB), human epidermal growth factor-2 gene (HER2), cathepsin D, etc.—are used as investigation tools in order to determine the most effective treatment method [2].

Breast conserving treatments such as chemotherapy, hormonal therapy, targeted therapy, immunotherapy and radiation therapy are recommended in the early stages of BC. On the other hand, mastectomy procedures (supported by classical therapeutic strategies) are increasing due to tumor microenvironment heterogeneity and aggressivity [3]. Considering these aspects, many studies to date have focused on discovering effective therapeutic alternatives to increase healing potential. Recently, the most popular approach has been to combine antitumor BC drugs (e.g., 5-fluorouracil, doxorubicin, docetaxel, paclitaxel, trastuzumab) with drug vehicles (e.g., nanobiomaterials, liposomes) [4].

5-Fluorouracil (5-FU) is an anti-cancer drug, widely used for cancer treatment [5,6,7,8] due to its significant therapeutic effects and reasonable purchase price. In this context, 5-FU may also be exploited as a potential anti-cancer drug in BC [9,10,11]. 5-FU’s mechanism of action is based on the inhibition of thymidylate synthase, which determines intracellular deoxynucleotide pools’ detachment and alters the mechanism of DNA replication [12]. Even though it is one of the most widely used anti-tumor drugs, 5-FU’s therapeutic efficacy can be enhanced through its incorporation into biomaterials or nanoparticles. This approach can be an efficient drug delivery strategy thanks to advantages such as high biocompatibility, biodegradation, and the possibility of decreasing the side effects of anti-cancer drugs [4].

In some cases, following surgical treatment, a method of breast soft tissue reconstruction is recommended. Adipose tissue engineering involves the regeneration or reconstruction of excised adipose tissue using scaffolds capable of supporting cell growth and proliferation. In this context, biomaterials’ research has been of significant interest within the biomedical domain. This is particularly true for scaffold design and fabrication, as they can act as synthetic frameworks for the integration of active ingredients or used to develop artificial materials that can induce the repair and restoration of damaged tissue [13].

One common strategy to tailor the release profile of active ingredients is the use of hydrocolloids as bio-based carrier vehicles for controlled drug delivery. This strategy has the advantages of hydrophilicity and biocompatibility. Scaffolds composed of natural polymers (cellulose, alginate, pectin, etc.) are preferred due to their high degree of porosity, resorption capacity or ability to mimic the extracellular matrix [13].

As such, there has been increased interest in renewable feedstock, among which starch, alginate (ALG), carrageenan’s and pectin (PEC) are the most well-known examples of natural hydrocolloid biopolymers with features deemed useful in pharmaceutical formulations [14]. Aside from their applications in pharmaceutics, the properties of hydrocolloidal formulations based on ALG or PEC render them useful in fields such as tissue engineering, cosmetics, and the food industry [15,16].

ALG and PEC are the main native constituents of plant cell walls, along with cellulose—which is the primary structural building block of plant structural components. ALG and PEC are sourced from marine (e.g., brown algae) and terrestrial (e.g., citrus, apple) plants, respectively. Nanocellulose—cellulosic material with its spatial dimensions reduced to nanoscale—may be isolated from various plant sources such as wood, cotton, wheat straw, and bamboo. Its properties vary widely depending on the source and extraction method. Interest in nanocelluloses has grown rapidly with the introduction and optimization of controlled oxidative treatments [17] that tune the surface properties and render gel-like suspensions of cellulose. Among them, oxidation mediated by 2,2,6,6-Tetramethyl-1-piperidinyloxy (TEMPO) generates individual negatively charged cellulose nanofibers (CNFs).

Polysaccharide-based hydrogels are considered fascinating biodegradable drug-delivery vehicles; for both ALG and PEC, ionotropic gelation in the presence of divalent cations (such as Ca^2+^) is highly convenient. Thus, it has become the most widely used crosslinking strategy for obtaining hydrogels as a matrix for the delivery or entrapment of various active ingredients [18]. Bacterial nanocellulose in combination with ALG supports cell differentiation, increases angiogenesis and cell proliferation and is indicated for tissue engineering and soft tissue reconstruction [19].

Human adipose-derived stem cells (hASCs) have been used in numerous regenerative medicine strategies due to their self-renewal capacity, low immunogenicity, high proliferative rate and potential to differentiate on multiple lineages (e.g., adipogenic, osteogenic, chondrogenic, myogenic, neurogenic) [20]. Many studies reported the ability of hASCs to differentiate toward the adipogenic lineage in vitro and in vivo, especially in association with nanobiomaterials, stimulating the regeneration of adipose tissue. These studies contributed to the development of post-mastectomy breast reconstruction methods, reducing the risk of contractile capsule occurrence, ruptures or infections [21].

Tumor microenvironments are associated with the development of inflammatory reactions. Chronic inflammation, supported by tumor microenvironment cells, promotes the activation of the inflammasome complex [22]. Inflammasomes are cytoplasmic multiprotein complexes consisting of three molecular structures: sensor molecule, adaptor protein and effector. After stimuli detection and activation of sensor molecules, the adaptor protein is oligomerized and caspase-1 is activated. Afterward, caspase-1 influences the cleavage and maturation of pro-interleukin (IL)-1β and pro-IL-18, promoting the inflammatory cascade and pyroptosis [23].

On the other hand, chemotherapeutic agents stimulate oxidative stress. 5-FU enhances oxidative stress in the cells through the generation of free oxygen radicals and induces damaging effects in the antioxidant defense mechanism. Reactive oxygen species (ROS) are often released by cells in response to chemotherapeutic drugs. Excessive ROS generation can cause cell death [24].

Many luminal BCs carry point mutations in p53. TP53 is a tumor suppressor, targeted by 5-FU, which triggers cell cycle blockage and programmed cell death. Additionally, P53 protein induces 5-FU-based cell death [25]. Previous studies indicated that administration of 5-FU treatment provoked dynamic activation of caspase-1 after 12 h [26]. Moreover, 5-FU-mediated caspase-1 activation leads to IL-1β and IL-18 secretion and pyroptosis initiation [27].

The present study utilized a complex approach, envisaging several directions: (1) a bioengineering approach studying the optimal parameters of blending CNFs with natural polysaccharides (alginate/pectin) and an anti-tumor agent (5-FU) in order to create an efficient, biocompatible three dimensional (3D) anti-cancer platform; (2) a drug delivery approach for breast cancer aiming to investigate the effects of 5-FU on breast cancer cells, ROS production and inflammasome activation; and (3) a tissue engineering approach studying the possibility of post-mastectomy soft tissue reconstruction based on human adipose-derived stem cells. The aim of this study was to investigate the potential of CNFs/5-FU systems to counteract breast cancer cell proliferation by inducing caspase-1 dependent cell death (pyroptosis) while supporting both normal breast cells’ and hASCs’ viability and proliferation in order to facilitate soft tissue reconstruction.

## 2. Materials and Methods

### 2.1. Materials

Alginic acid sodium salt from brown algae (low viscosity) was acquired from Sig-ma-Aldrich (Steinheim, Germany) and used as such. High-methoxylated pectin, extracted from apple with an esterification degree of 70–75%, was purchased from Sigma-Aldrich and subjected to chemical modification in order to yield low-methoxylated pectin according to the protocol described in our previous report [28]. Aqueous CNFs (1.13% *w*/*v*) with a total acidic content of 835 µmol/g were prepared from never-dried, bleached-kraft pulp from softwood (kindly supplied by StoraEnso^TM^ (Stockholm, Sweden) according to our previous report [29], using the TEMPO/NaBr/NaClO at pH 10.5, also in agreement with a previous study with NaClO of 2.5 mmol g^−1^ cellulose [17].

The reagents employed in the modification treatment of cellulose and HM-pectin, re-spectively, such as TEMPO (free radical, 98%), sodium bromide ACS (≥99%), sodium hy-pochlorite solution (NaClO) (12% active chlorine) EMPLURA^®^, sodium hydroxide 98% (pellets) and hydrochloric acid ACS (37%), were reagent-grade chemicals purchased from Sigma-Aldrich (Steinheim, Germany). Calcium chloride ≥ 97.0% (anhydrous, free flowing, Redi-Dri™) and phosphate-buffered saline (PBS) (as powder, yielding 0.01 M PBS, 0.138 M NaCl and 0.0027 M KCl, with a pH of 7.4) were also purchased from Sigma-Aldrich (Steinheim, Germany).

Dulbecco’s Modified Eagle’s Medium (DMEM), RPMI-1640 medium, antibiotic antimycotic solution, phosphate-buffered saline (PBS) powder, Cholera toxin from Vibrio cholerae supplement, MTT assay kit for quantitative evaluation of viability, Tox7-KT LDH assay kit for evaluation of cytotoxicity, DCF-DA (D6883), Ampliflu Red (90101), bovine serum albumine (BSA), Triton-X100, paraformaldehyde (PFA) solution, phalloidin-FITC and Hoechst 33,258 solution for fluorescence staining of actin filaments and nuclei were purchased from Sigma-Aldrich, Steinheim, Germany. Fetal bovine serum (FBS) and Live/Dead assay kit were purchased from Thermo Fisher Scientific, Waltham, MA, USA. Three cell lines—MDA/MB 231 (HTB-26), ZR 75-1 (CRL-1500) and MCF12A (CRL-10317)—were purchased from ATCC, Manassas, USA. MEBM Basal Medium and MEGM Supplement Pack were purchased from Lonza/Clonetics Corporation, Basel, Switzerland. TRIzol Reagent, goat anti-mouse secondary antibody AlexaFluor 546 (A11003), and goat anti-rabbit secondary antibody AlexaFluor 546 (A11008) were purchased from Invitrogen, Waltham, MA, USA. p53 (sc-126) and caspase-1 antibodies (2225S) were purchased from Cell Signaling Technology, Inc., Danvers, MA, USA. Caspase-Glo^®^ 1 Inflammasome Assay kit was purchased from Promega, Madison, WI, USA, and iScript cDNA Synthesis kit was purchased from BioRad, Hercules, CA, USA.

### 2.2. Preparation of Polymeric Scaffolds

The strategy used to obtain polymeric scaffolds was based on combining two types of poly-saccharides to achieve materials with synergistic properties. Materials based on ALG/CNFs and PEC/CNFs were obtained by dispersing the heteropolysaccharide (ALG or PEC) within the CNFs gel in a ratio of 3:1. The solution was magnetically stirred for 15 min and different amounts of 5-FU were added in order to get two distinct concentrations: 1 and 5 mg/mL. Hydrogel samples were prepared via crosslinking by immersion in CaCl_2_ solution (3%, 10 min) and subsequently, the porous structure was attained by freeze-drying. The tabulated summaries of the hydrogel compositions and sample codes are described in Table 1.

### 2.3. Fourier Transform Infrared (FTIR) Spectrometry

The structural features of the materials were evaluated primarily by Fourier Transform Infrared (FTIR) measurements using a Vertex 70 Bruker FTIR spectrometer equipped with an attenuated total reflectance (ATR) cell with Ge crystal. In all cases, the FTIR spectra were recorded at room temperature using 32 scans in wavelengths ranging from 600–4000 cm^−1^, 4 cm^−1^ resolution.

### 2.4. Micro-CT Analysis

Micro-architectural particularities/features of the polymer blend substrates were surveyed via computer assisted microtomography (µCT). The size, geometry and overall interconnectivity of the freeze-dried samples were targeted. A Bruker µCT 1172 high-resolution micro-computer tomography scanner was used to analyze one rectangular (1.5 × 5 mm) sample of each composition. The specimens were mounted on the scanning stage without further treatment and scanned at invariable/constant parameters: a source voltage of 50 kV and a current intensity of 175 mA. Throughout, datasets were acquired in 3 h, during 180° rotations of the specimen, with a rotation step of 0.1° and frame exposure of 800 ms. Each slice was averaged from 5 successive acquisitions. The resolution of each 2D projection was 2452 × 1640 pixels and the image pixel sizes were fixed at 1.5 µm. Raw data reconstructions were performed in Bruker NRecon 1.7.1.6 software, 2015 (Bruker, Kontich, Belgium). Reconstructed tomograms were depicted in 3D in Bruker CTVox, version 3.3.0r1403, 2017 (Bruker, Billerica, MA, USA).

### 2.5. Swelling Behavior

The swelling behavior of the porous materials was evaluated in PBS. Out of each composition, three specimens of known weights (*W_d_*) were placed in PBS of pH 7.4 for 24 h at room temperature. Thereafter, samples were drowned out of PBS and filter paper was used to remove water on the surfaces of the samples. The wet weight of the samples (*W_s_*) was recorded and the maximum swelling degree (MSD %) for each sample was calculated according to the following equation:

(1) $$\mathrm{MSD}\;\left(\%\right)=\frac{W_{s}-W_{d}}{W_{d}}\cdot 100$$

The average values were reported with error bars depicting standard deviation.

### 2.6. Cell Culture and Subcultivation

For the purpose of this experimental set up, 4 cell types were used: a human breast adenocarcinoma cell line (MDA/MB 231), a ductal carcinoma cell line (ZR 75-1), a normal control represented by mammary gland cell line (MCF12A) and hASCs obtained from liposuction (with patient written consent and approval of the Ethics Committee of University of Bucharest (protocol No.153/24.08.2017), in compliance with the Declaration of Helsinki.

MDA/MB 231 and hASCs cells were maintained and grown in DMEM enriched with 10% FBS and 1% antibiotic antimycotic solution. ZR 75-1 cells were cultured using RPMI-1640 medium enriched with 10% FBS, while MCF12A cells were cultured in the MEBM base medium for this cell line along with the additives from MEGM kit, supplied with 100 ng/mL cholera toxin and 10% FBS. All cell cultures were incubated in standard culture conditions—37 °C and 5% CO_2_ in a humidified atmosphere.

### 2.7. Drug Release Study

5-FU encapsulation efficiency within polymeric scaffolds was registered with a Cary 60 Agilent spectrophotometer equipped with a quartz cell having a light path of 10 mm. The UV spectra of CaCl_2_ solution in which the crosslinking was made were registered at λ = 266 nm.

The encapsulation efficiency (EE%) was calculated using Equation (2).

(2) $$\mathrm{EE}\%\;=\frac{m_{0}-m}{m_{0}}\cdot 100$$

where *m*_0_ is the initial 5-FU amount (g), *m* is the unloaded 5-FU amount (g).

The drug release profile was determined in an automated dissolution USP Apparatus 1 (708-DS Agilent) with an automatically controlled multichannel peristaltic pump (810 Agilent), a UV/Vis spectrophotometer (Cary 60) with 1 mm flow cell and UV-Dissolution software. The samples were immersed in 200 mL of dissolution medium (PBS) at 37 °C and monitored for 24 h. At specific time intervals, the amount of released 5-FU was determined with a known concentration of the standard solutions at 266 nm.

### 2.8. Achievement of 3D Biosystems

The 3D scaffolds (A.CNF.0 and P.CNF.0 enriched with 5-FU) were cut and sterilized by exposure to UV light. Next, cells were seeded at 2 × 10^5^ cells/cm^2^ for hASCs and MCF 12A cell lines and at 3 × 10^5^ cells/cm^2^ for MDA/MB 231 and ZR 75-1 cell lines. This resulted in a 3D biosystem, which was then incubated for one week in standard culture conditions. In contrast to 3D spheroids, which represent scaffold-free 3D cultures, the 3D biosystems analyzed in this study were hybrids obtained by cell seeding in 3D scaffolds, resulting in cell-scaffold constructs designed for tissue engineering approaches. Biocompatibility assessment was accomplished at 2 and 7 days post-seeding, gene expression studies for *p53* and caspase-1 were carried out at 12 and 24 h post-seeding, together with Caspase-Glo^®^1 inflammasome assay, reactive oxygen species’ (ROS) measurement and protein expression evaluation for P53 and caspase-1.

### 2.9. In Vitro Biocompatibility Evaluation of 3D Biosystems

For the evaluation of cell viability and proliferation, quantitative methylthiazolyldiphenyl tetrazolium bromide (MTT) assay was performed. The recommended working concentration for MTT was used, specifically 1 mg/mL in culture media lacking FBS. After discharging the culture media, the constructs were incubated with MTT solution for 4 h in standard conditions. The obtained formazan crystals were solubilized with isopropanol and the absorbance of the final product was measured at 550 nm using a FlexStation 3 Spectrophotometer (Molecular Devices, San Jose, CA, USA).

In order to establish the cytotoxicity of the materials upon cells, in vitro toxicology assay (lactate dehydrogenase-based) TOX7 kit was utilized. The assay was performed following manufacturer’s instructions and the final solution was measured at 490 nm, using a FlexStation 3 Spectrophotometer (Molecular Devices, San Jose, CA, USA). 

During quantitative biocompatibility studies, A.CNF- and P.CNF-enriched composites were compared to simple A.CNF.0 and P.CNF.0, considered controls. A separate bidimensional control of cells cultured on the plate surface (tissue culture plate (TCP)) was also considered as reference. In addition, a 5-FU-treated cells’ reference was also included in the study.

Live/Dead staining was performed using Live/Dead kit. The solution was prepared following manufacturer’s instructions and the scaffolds were incubated for 1 h in dark conditions. The visualization of live (green-labeled) and dead (red-labeled) cells was performed using a laser-scanning confocal microscope (Nikon A1/A1R Confocal Laser Microscope System, NY, USA) and images were analyzed using corresponding software. Quantification of the fluorescence both for live and for dead cells was obtained using ImageJ software.

### 2.10. Caspase-1 Activity Assay

In order to evaluate inflammasome complex activity, activated caspase-1 was directly monitored and selectively measured. Caspase-1 activity can be measured using the Caspase-Glo^®^ 1 inflammasome assay, a homogeneous and bioluminescent method. The principle of this method is based on aminoluciferin cleavage by caspase-1 and generation of light by recombinant luciferase, which generate stable luminescent signals directly proportional to caspase-1 activity. The assay was performed following manufacturer’s indications and the luminescent signals were measured at 12 h and 24 h using FlexStation 3.

### 2.11. Detection of Intracellular/Extracellular Reactive Oxygen Species (ROS)

#### 2.11.1. Intracellular ROS Production

The level of intracellular ROS was measured by using the fluorescent probe 2,7-dichlorofluorescin diacetate (DCFH-DA), which passively enters cells and reacts with ROS to form the highly fluorescent compound dichlorofluorescein (DCF). Briefly, the MCF12A, MDA/MB 231, and ZR 75-1 cells were seeded in 96-well black, sterile clear-bottomed plates at a density of 3 × 10^4^ cells/mL. After 24 h, the cells were incubated with 50 μM DCF-DA prepared in HBSS (Hank’s Balanced Salt Solution) solution, for 1 h at 37 °C and 5% CO_2_. Next, the cells were treated with P.CNF.0, P.CNF.1 and P.CNF.5 extracts for 6 and 24 h, respectively. The formation of fluorescent DCF was recorded using a microplate spectrofluorometer (FlexStation 3, Molecular Devices) at excitation and emission wavelengths of 485 nm and 520 nm, respectively. The level of ROS in treated samples was normalized to the cell number and expressed in percentages comparing to control.

#### 2.11.2. Extracellular H_2_O_2_ and H_2_O_2_ Efflux

Hydrogen peroxide (H_2_O_2_) detection was performed using a spectrophotometric assay based on horseradish peroxidase (HRP) in combination with Ampliflu Red (*N*-acetyl-3,7-dihydroxyphenoxazine). The last was oxidized by the hydroxyl radical (HRP catalyzes the decomposition of H_2_O_2_) into resorufin, a pink-colored compound. The assay used can detect extracellular H_2_O_2_ and H_2_O_2_ released from the cell [30]. To measure H_2_O_2_ levels, the MCF12A, MDA/MB 231, and ZR 75-1 cells were seeded in 96-well plates at a density of 3 ×10^4^ cells/mL. After 24 h, the cells were incubated with P.CNF.0, P.CNF.1 and P.CNF.5 extracts. Immediately after treatment, a volume of 100 µL/well freshly prepared reaction mix, containing 0.1 mM Ampliflu Red, 2 mM NaN3, and 2 U/mL HRP in Krebs Ringer’s Phosphate Glucose (KRPG) buffer, pH 7.4, was added. The plates were incubated at 37 °C in the dark for 6 h and 12 h, respectively. The optical density was read at 570 nm. Background absorbance, determined at 620 nm, was subtracted from each correspondent value. The sample values were normalized to control and results were expressed as percentages. A 30 µM solution of H_2_O_2_ was used as a positive control.

### 2.12. Quantitative PCR (qPCR)

Next, p53 and caspase-1 gene expression was analyzed by qPCR, considering a comparison between their levels in normal and tumor cells exposed to control scaffold (P.CNF.0) or 5-FU-enriched scaffolds (P.CNF.1, P.CNF.5). The bioconstructs obtained were cut into fragments and the total RNA was isolated using TRIzol Reagent according to the manufacturer’s indications. Total RNA was tested for purity and concentration using NanoDrop spectrophotometer (ThermoScientific, Waltham, MA, USA) and RNA integrity number (RIN) was determined using Agilent 2100 BioAnalyzer (Agilent Technologies, Waldbronn, Germany). Next, total cellular RNA was reverse-transcribed to complementary DNA (cDNA) using iScript cDNA Synthesis kit. The gene expressions of p53 and caspase-1 were evaluated by qPCR using SYBR Green method and ViiA 7 equipment (ThermoScientific, Waltham, MA, USA). Every sample was evaluated in triplicate, and the expression of GAPDH was used as reference gene. Sequences of gene-specific primers used were: GAPDH sense 5′-AAGGTCGGAGTCAACGGATT-3′, GAPDH antisense 3′-CTCCTGGAAGATGGTGATGG-5′, p53 sense 5′-CGAGATGTTCCGAGAGCTGAAT-3′, p53 antisense 3′-TTTATGGCGGGAGGTAGACTGA-5′, caspase-1 sense 5′-GCCTGTTCCTGTGATGTGGAG-3′ and caspase-1 antisense 3′-TGCCCACAGACATTCATACAGTTTC-5′.

### 2.13. Immunofluorescence

Caspase-1 and p53 protein expression were investigated by immunostaining and confocal microscopy. Bioconstructs were fixed with a 4% paraformaldehyde solution for one hour and permeabilized with 0.1% Triton X100 solution in 2% BSA for 20 min, at 4 °C. Next, the bioconstructs were incubated overnight with mouse monoclonal antibody p53 and rabbit polyclonal antibody caspase-1, and then with goat anti-mouse secondary antibody AlexaFluor 546 and goat anti-rabbit secondary antibody AlexaFluor 546, respectively, for 1 h at 4 °C. F-actin filaments were stained using -phalloidin-FITC according to manufacturer’s instructions. Cell nuclei were stained with Hoechst 33,258 solution. All the images were visualized and captured using a confocal microscope (Nikon A1/A1R Confocal Laser Microscope System). Quantification of the fluorescence levels for p53 and caspase-1 in the captured images was performed using ImageJ software.

### 2.14. Statistical Analysis

All performed experiments were carried out in triplicate (*n* = 3) and the generated results were expressed as means ± standard deviation using GraphPad Prism 6.0 Software (GraphPad Software Inc., version 6.01, 2012, San Diego, CA, USA). The statistical relevance was assessed using the same software by one-way ANOVA method and Bonferroni post-test, considering a statistical difference for *p* < 0.05.

## 3. Results

### 3.1. Fourier Transform Infrared (FTIR) Spectrometry

**Figure 1:**
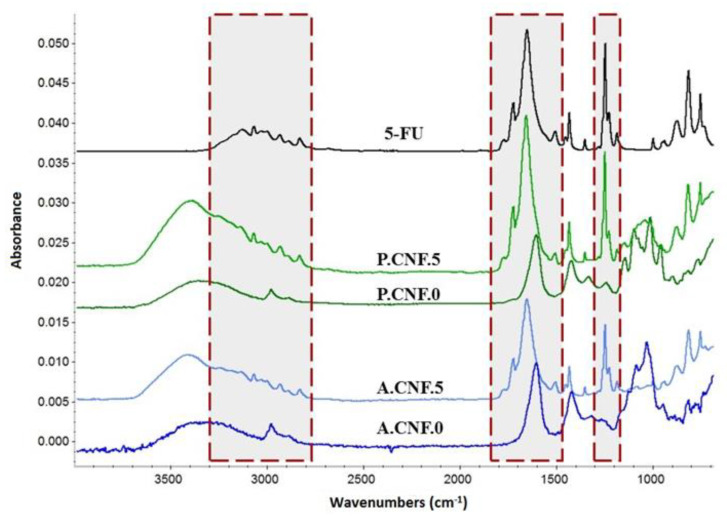
Influence of 5-FU on the FTIR spectra of the ALG- and PEC-based materials.

FTIR spectrometry was employed in furtherance of materials’ structural characterization; the spectra peculiarities are discussed below. The FTIR data of the Ca^2+^-crosslinked porous bio-platforms resulted from overlapping the absorption spectra from biopolymer constituents and 5-FU. Figure 1 illustrates the FTIR spectra of bare and high concentration drug-loaded polysaccharidic materials. To highlight the presence of 5-FU’s specific signals, the FTIR spectrum of the therapeutic drug is displayed.

In the FTIR spectra of cross-linked nanofibrillar scaffolds, the characteristic absorption bands of CNFs, overlapped with those of ALG or PEC, were observed. The broad band widening in the 3000 to 3600 cm^−1^ range was assigned to OH bending. Two small peaks that appeared around 2940 and 2870 cm^−1^ were attributed to CH asymmetric and symmetric stretching vibration and signals at around 1100 and 1030 were related to the C–O and C‒O‒C vibration of pyranose rings. Carboxylate C-O stretching generated an intense signal that could be observed in the 1600–1630 cm^−1^ range for all polysaccharides. Peaks provided by 5-FU were recorded in between 3100 and 2900 cm^−1^ and attributed to –NH groups. The carbonyl groups’ signal appeared at 1650 cm^−1^, while the characteristic adsorption band of C‒F vibration was registered at 1245 cm^−1^.

Upon comparing the FTIR spectra of the pure drug and the drug-containing hydrogels, no significant shifting of signals was observed, revealing that the selected crosslinking strategy did not impact the active molecule.

### 3.2. Micro-CT Analysis

**Figure 2:**
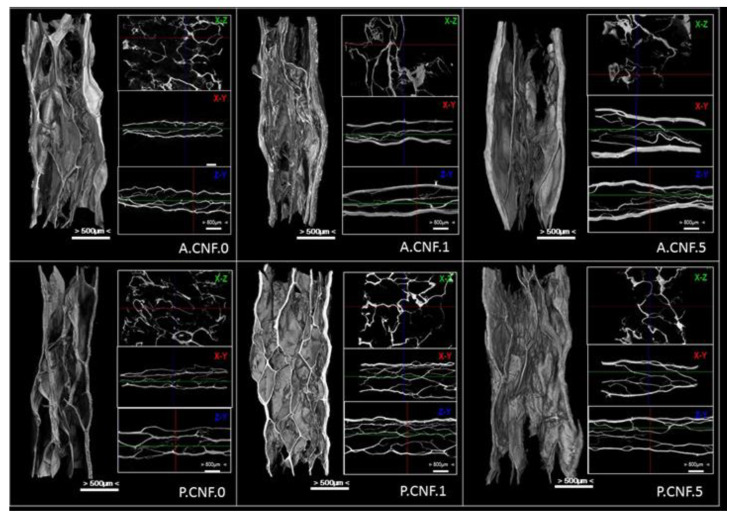
MicroCT images of dried nanofibrillar hydrogels.

Figure 2 shows representative Micro-CT images of nanofibrillar composite materials with aligned macroporous structures which were successfully prepared by freeze-drying. 

Porous structures with interconnected pores of irregular shapes were observed in the hybrid hydrogel. The images provided direct evidence that the CNFs could be well dispersed in ALG or PEC matrix.

For both ALG- and PEC-based materials, aligned micro-channels within nanofibrillar hydrogels formed as ice crystals, and the strong interactions between the hydrocolloid chains rendered its ability to sustain itself after freezing. More importantly, CNFs with many oxygen-containing functional groups can be well dispersed in ALG/PEC matrix due to strong H-bonding between nanofibers and the polymer chains. After freezing, the side surface of the gel sample exhibited the well-defined directional structure and the width of the aligned channels varied from 100–350 μm. One major reason was that the high water content of the hydrogel sample was employed (95 wt%) during the freeze-drying process, in which a large number of ice crystals were removed and the amount of ALG/PEC chains built a 3D micropore structure to form the gel. On the other hand, the large nanofibrils of cellulose exhibit the ability to sustain the inner structure of hydrogel. However, it proved proven difficult to observe the self-standing CNFs in the Micro-CT images, which can be attributed to the large numbers of ALG or PEC chains dispersed into the polymer matrix covering or entangling the nanofibers.

With the addition of 5-FU, a much tighter microporous structure was observed, as were thicker walls. Microstructural analysis of biomaterials showed the important details of the morphology, and the derived quantitative and qualitative information was maximized. The interconnection of the pores was an important parameter; it defined the effects of porosity and pore size. This parameter was observable in cross-sectional 2D images in the transverse planes (x-y, x-z and z-y)

The internal architecture of a scaffold plays a major role in cell behavior—and thus on the overall performance of the construct. The characterization of its macro- (>100 μm) and micro- (0.1–100 μm) structure is thus inherently of interest.

### 3.3. Swelling Behavior 

**Figure 3:**
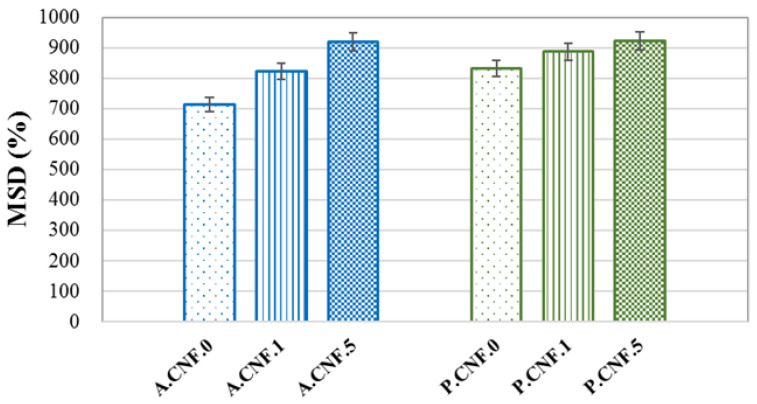
Maximum swelling degree (MSD) of porous nanofibrillar hydrogels.

To estimate the MSD, the samples were monitored for 24 h at time, during which it was observed that the materials remained stable. In addition, a fast water adsorption was noted within the first minutes of immersion, while equilibrium was reached in about 15 min. Figure 3 depicts the MSD values for the synthetized materials.

High swelling ratios were attained by all samples on account of the high hydrophilic character of both ALG and PEC. The addition of 5-FU seemed to enhance water absorption, leading to an increase in MSD as the amount of drug increased. This phenomenon was correlated with morphological features, in which the porosity was higher for drug-loaded materials. Moreover, it was observed that the PEC-based hydrogels exhibited a slightly higher swelling capacity with respect to ALG matrices.

### 3.4. In Vitro Biocompatibility Evaluation of 3D Biosystems

**Figure 4:**
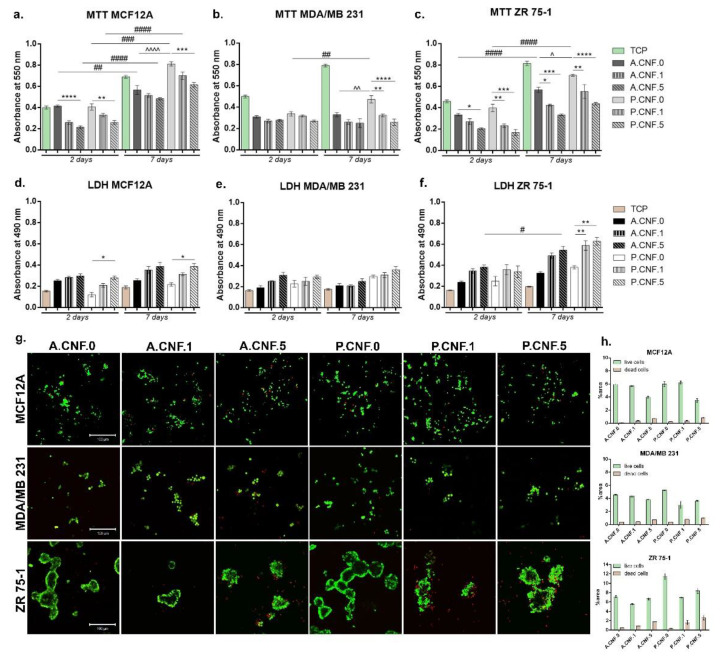
Biocompatibility assessment of A.CNF.0- and P.CNF.0-based scaffolds in contact with MCF12A, MDA/MB 231 and ZR 75-1 breast cells. (**a**–**c**) Cell viability and proliferation profiles obtained after 2 and 7 days of culture in standard conditions by MTT assay. Statistical significance: * *p* < 0.05, ** *p* < 0.01, *** *p* < 0.001, **** *p* < 0.0001, ## *p* < 0.01, ### *p* < 0.001, #### *p* < 0.0001, ^ *p* < 0.05, ^^ *p* < 0.01, ^^^^ *p* < 0.0001. (**d**–**f**) A.CNF.0- and P.CNF.0-based scaffolds’ cytotoxicity evaluation, as revealed by LDH assay after 2 and 7 days of culture. Statistical significance: * *p* < 0.05, ** *p* < 0.01, # *p* < 0.05. (**g**) Qualitative Live/Dead staining, showing live cells (green) and dead cell nuclei (red) of MCF12A, MDA/MB 231 and ZR 75-1 cells in contact with A.CNF.0- and P.CNF.0-based scaffolds. Scale bar 100 μm. (**h**) Quantification of green fluorescence (live cells) levels and red fluorescence (dead cell nuclei) levels in all composites.

After performing MTT assay, cell viability profile (Figure 4) indicated at 2 days post-seeding that normal cells (MCF12A) on the control scaffold (A.CNF.0) presented a significantly higher viability (*p* < 0.0001) in comparison to A.CNF.5. A similar cellular response was observed on P.CNF.0 scaffolds, where viability on the control was significantly higher (*p* < 0.01) than on P.CNF.5. After 7 days of culture, no relevant differences were noted between A.CNF.0 based scaffolds, whereas on P.CNF.0 scaffolds, a significantly higher (*p* < 0.001) cell viability was observed on the tested P.CNF.0 control, as compared to P.CNF.5. Of note: on P.CNF.0 materials, the quantity of metabolic active cells was significantly higher (*p* < 0.0001) than on A.CNF.0 materials. During one week of culture, normal cells had a significant proliferation profile in contact with the controls.

Quantitative MTT assay for MDA/MB 231 cell lines revealed overall low cell viability in contact with all tested composites. After 2 days of culture, no significant differences were observed between materials. This changed after 7 days of culture, but only in the case of P.CNF.0 materials, where it was observed that the viability of MDA/MB 231 cells was significantly higher than P.CNF.1 and P.CNF.5 (*p* < 0.01 and *p* < 0.0001 respectively). In the case of the MDA/MD 231 cell line, significantly higher (*p* < 0.05) viability was found on the P.CNF.0 control, in contrast to the A.CNF.0 control. Concerning the other tumor cell line, ZR 75-1, MTT profile at 2 days post-seeding showed significant cell viability (*p* < 0.05) on A.CNF.0 control as compared to A.CNF.5. A better rate of cell viability was also found in contact with P.CNF.0 control than that observed with P.CNF.1 and P.CNF.5 (*p* < 0.01 and *p* < 0.001 respectively). Within 7 days of culture, the highest level (*p* < 0.0001) of live cells was found on the P.CNF.0 control, as compared to the other two materials enriched with 5-FU. As for the A.CNF.0 material, observation revealed that the control cells were found to possess significantly higher (*p* < 0.01 and *p* < 0.001) metabolic activity than A.CNF.1 and A.CNF.5. It is of note that the viability on P.CNF.0 scaffolds was significantly better (*p* < 0.05) than on the A.CNF.0 scaffold, as this was also observed in case of the ZR 75-1 tumor cell line. Cells proliferated significantly (*p* < 0.0001) over the period from 2 to 7 days of in vitro cell culture, especially on A.CNF.0 and P.CNF.0 tested controls. 

Overall, LDH testing indicated that the 3D fibrillary scaffold induced certain levels of toxicity upon the chosen cell types, which were significantly higher on the materials which contained 1 and 5 mg/mL concentrations of 5-FU. MCF12A cytotoxicity profile indicated that during one week of cell culture, relatively low levels of LDH were found in contact with these materials. At 2 and 7 days post-seeding, a significantly high (*p* < 0.05) mortality rate was found on P.CNF.5. Aside from this change, the quantities of dead cells did not change significantly from 2 to 7 days of culture. 

In the case of the MDA/MB 231 tumor cell line, there were no significant differences in toxicity between the tested composites. Even so, taking into consideration the levels of Live/Dead revealed by MTT assay, the levels of LDH were not unexpected. In the case of those tumor cells, the addition of a higher concentration of 5-FU did not have a significant impact on cell behavior. For the ZR 75-1 tumor cell line, no significant differences were observed after 2 days of culture; however, after 7 days, the levels of LDH were higher, with a significant level (*p* < 0.05) of LDH registered on the A.CNF.5 scaffold. Most significantly, (*p* < 0.01) levels of toxicity were found on P.CNF.1 and P.CNF.5, as compared to the P.CNF.0 control. 

The fluorescent images obtained after performing Live/Dead staining revealed the same results that were obtained after carrying out MTT and LDH assays. After 7 days of cell culture, normal breast cells formed groups on A.CNF.0 scaffolds, but more green-labeled cells were identified on P.CNF.0 materials. Small amounts of dead cells were observed on all biosystems.

In the case of MDA/MB 231 tumor cells, the same phenotype was observed on all tested composites, but a higher amount of red-labeled cells were noted on A.CNF.5 and P.CNF.5. Live/Dead staining confirmed that cells proliferated only on P.CNF.0 control; on the other materials, no significant proliferation was observed. As for the ZR 75-1 cell line, on the controls A.CNF.0 and P.CNF.0, cells formed large groups with few dead cells. This was in contrast to the materials which contained 5-FU, in which cells were found in smaller groups, surrounded by larger amounts of red-labeled cells. Overall, A.CNF.0/P.CNF.0 materials displayed biocompatibility with normal cells, while the addition of 1 and 5 mg/mL 5-FU induced significant cytotoxicity in tumor cells.

### 3.5. Drug Release Study

Measurements for determining EE% were carried out using UV absorbance at varying concentrations. Based on the UV absorbance and Equation (2), the EE was estimated at 62 ± 2.3% for A.CNF.1, 68 ± 3.7% for A.CNF.5, 72 ± 3.3% for P.CNF.1 and 76 ± 2.8% for P.CNF.5. The chain structure of the polymeric network contained several hydroxyl, carboxyl and carbonyl groups. These facilitated the formation of van der Waals interactions with electronegatively charged molecules, like fluorine, in 5-FU. The mechanical entrapment of the drug within the polymerics matrices resulted in a high EE value. The EE increased along with the amount of drug added and the samples with PEC were able to encapsulate with about 10% more. 

**Figure 5:**
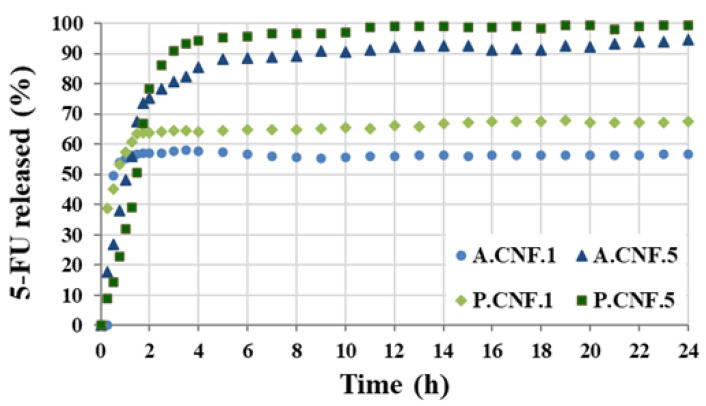
Drug release percentage of porous nanofibrillar hydrogels in PBS at pH 7.4.

The drug release pathways differed according to a number of variables, including the scaffold morphology and pore dimensions, the type of drug/polymer(s) interactions and the scaffold degradation rate. Figure 5 displays the release profiles of 5-FU from the hydrogels in PBS at 37 °C. 

During the first hour, all samples showed a burst release. This accelerated initial re-lease was ascribed to the existence of unencapsulated drug or to the proximity of drug molecules to the surface. Although both A.CNF.1 and P.CNF.1 released about 55% of the loaded drug within 1 h, the initial burst decreased with increasing 5-FU content. This was more evident for PEC-containing scaffolds as, by the same time, P.CNF.5 released only 31% of drug. As illustrated in the graph, it was evident that, for A.CNF.1 and P.CNF.1, the release occurred within 4 h and reached maximum percentages of 56 ± 2.8 and 67 ± 1.3, respectively, during the 24 h. A marked difference between samples was evident when different quantities of 5-FU were loaded: for A.CNF.5 and P.CNF.5, the drug was almost completely released from the matrix over a period of 4 h. Clearly, the amount of 5-FU released increased over time, giving overall releases of about 94% for A.CNF.5 and 99% for P.CNF.5. In correlation with Micro-CT analysis and swelling tests, these results could be explained as a chain effect in which the addition of the drug induced changes in the morphology and swelling capacity of the scaffolds. Based on a more porous network and higher swelling degree, the matrix density decreased, thus facilitating the escape of 5-FU molecules [31]. Furthermore, both the EE% and the percentage of drug released were found to be significantly higher in the PEC hydrogels than in the ALG samples. Based on the number of free acid groups, PEC had lower gel strength than ALG; thus, the weaker the gel barrier, the higher the percentage of released drug [32]. The results showed that PEC-containing hydrogels could be a better formulation for controlled drug release matrices.

### 3.6. Caspase-1 Activity Evaluation

**Figure 6:**
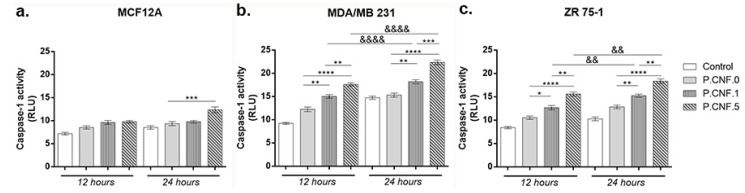
Caspase-1 activation in (**a**) MCF12A, (**b**) MDA/MB 231 and (**c**) ZR 75-1 cells in contact with P.CNF.0, P.CNF.1 and P.CNF.5 for 12 h and 24 h. Statistical significance: * *p* < 0.05, ** *p* < 0.01, *** *p* < 0.001, **** *p* < 0.0001, && *p* < 0.01, &&&& *p* < 0.0001.

Inflammasomes are cytoplasmic multiprotein complexes activated by inflammatory stimuli that modulate caspase-1, resulting in the maturation and release of IL-1β, IL-18 and pyroptosis. Using a bioluminescent method, caspase-1 activity was detected in breast cells following contact with P.CNF.0, P.CNF.1 and P.CNF.5 (Figure 6).

Slightly ascending but statistically insignificant profiles of caspase-1 activity suggested the cytotoxic effect of the anti-tumor agent embedded in P.CNF-enriched scaffolds on MCF12A normal breast cells. After 24 h the highest levels of caspase-1 activity were detected in contact with P.CNF.5, in comparison with P.CNF.0 (*p* < 0.001).

In the case of the MDA/MB 231 tumor cell line (Figure 6), after 12 h of incubation, there were no significant differences of caspase-1 levels between the control and P.CNF.0. However, the contact of tumor cells with P.CNF.1 and P.CNF.5 led to a significant increase in caspase-1 production, compared with P.CNF.0 (*p* < 0.01 and *p* < 0.0001). After 24 h of incubation, 5-FU presence in P.CNF-derived scaffolds enhanced caspase-1 production in culture media in a dose-dependent manner. Additionally, after 24 h of incubation, caspase-1 levels were increased in contact with P.CNF.5, in comparison with P.CNF.1 (*p* < 0.01), suggesting that the highest concentration of 5-FU added in the scaffolds’ composition caused the acceleration of caspase-1 production. Likewise, the contact of tumor cells with P.CNF.1 and P.CNF.5, respectively, induced significantly increased (*p* < 0.01 and *p* < 0.0001, respectively) caspase-1 levels in a time-dependent manner.

ZR 75-1 cancer cells released lower levels of caspase-1, compared with MDA/MB 231 cancer cell, suggesting the aggressiveness of triple negative BC. After 12 h, ZR 75-1 cells released higher capase-1 levels, compared with control. P.CNF.1 and P.CNF.5 exhibited significantly increased caspase-1 activity, compared with P.CNF.0 (*p* < 0.05 and *p* < 0.0001). Additionally, higher concentrations of 5-FU embedded in P.CNF.5 caused a significant increase (*p* < 0.01) in caspase-1 levels, compared to P.CNF.1. After 24 h of incubation, the most significant (*p* < 0.01 and *p* < 0.0001) levels of caspase-1 were found on P.CNF.1 and P.CNF.5, as compared to P.CNF.0 control. The same ascending profile of the caspase-1 levels (determined by the increase in 5-FU concentration) was observed between P.CNF.1 and P.CNF.5 (*p* < 0.01). In addition, increased incubation time allowed for significantly higher caspase-1 activity in contact with P.CNF.1 and with P.CNF.5 (*p* < 0.01).

### 3.7. Intracellular/Extracellular Reactive Oxygen Species (ROS) Measurement

**Figure 7:**
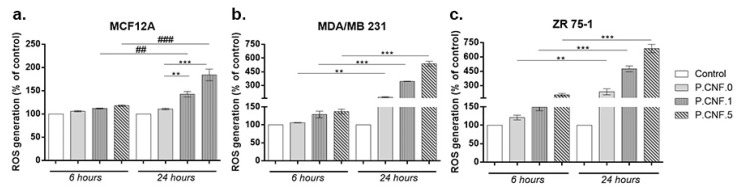
Level of ROS generated in the presence of P.CNF.0, P.CNF.1 and P.CNF.5 after 6 and 24 h of exposure in (**a**) MCF12A, (**b**) MDA/MB 231 and (**c**) ZR 75-1 cells. Statistical significance: ** *p* < 0.01, *** *p* < 0.001, ## *p* < 0.01, ### *p* < 0.001.

The ROS production was measured upon treatment in order to estimate the potential abilities of P.CNF.0, P.CNF.1 and P.CNF.5 to induce oxidative stress in breast cells. Thus, both the intracellular concentrations of ROS and the levels of H_2_O_2_ outside the cells were analyzed. As illustrated in Figure 7, exposure to P.CNF.1 and P.CNF.5 led to a significant elevation of intracellular ROS level that was time-dependent in all tested cell lines, as compared to unexposed cells. However, the increase was approximatively 2-fold higher after 24 h in cancerous MDA/MB 231 and ZR 75-1 cells, as compared to normal MCF12A breast cells. Moreover, it was found that exposure to P.CNF.0 induced significant generation of ROS, but only in breast cancer cells. The highest intracellular ROS level was observed in cells exposed to P.CNF.5 and the lowest level of ROS was registered in cells treated with P.CNF.0. Furthermore, we noticed that P.CNF.1 and P.CNF.5 induced a higher level of ROS in ZR 75-1 cells than others, probably due to a less efficient antioxidant system.

**Figure 8:**
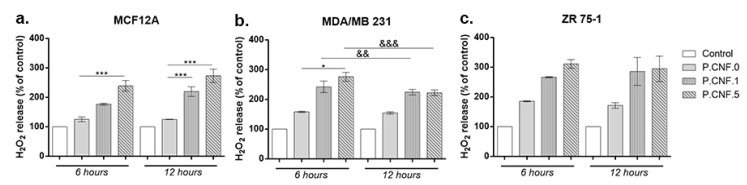
Rate of H_2_O_2_ production, as measured by Amplex Red, in (**a**) MCF12A, (**b**) MDA/MB 231 and (**c**) ZR 75-1 cells after contact with P.CNF.0, P.CNF.1 and P.CNF.5 scaffolds. Statistical significance: * *p* < 0.05, *** *p* < 0.001, && *p* < 0.01, &&& *p* < 0.001.

The extracellular H_2_O_2_ level is presented in Figure 8. The results showed a significant increase of H_2_O_2_ concentration in culture media of all types of breast cells treated with P.CNF.1 and P.CNF.5 starting with 6 h of exposure, as compared with unexposed cells and P.CNF.0. The increase was dependent on the concentration of 5-FU from tested materials in normal and cancer cells after 6 h of exposure. Nevertheless, after 12 h, the level of H_2_O_2_ was similar for breast cancer cells exposed to P.CNF.1 and P.CNF.5, but not for normal cells. Similarly to the results described above regarding intracellular ROS, the P.CNF.0 material caused a significant accumulation of H_2_O_2_, but only in culture media of breast cancer cells. In accordance with the intracellular ROS production, we found the highest levels of H_2_O_2_ in media of ZR 75-1 cells. The H_2_O_2_ level found in media of MDA/MB 231 cells was quite similar to that of normal cells. 

### 3.8. Gene Expression Evaluation

**Figure 9:**
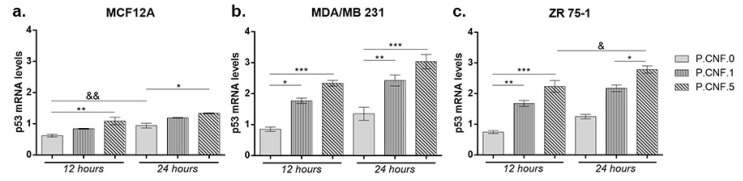
Evaluation of p53 gene expression in (**a**) MCF12A, (**b**) MDA/MB 231 and (**c**) ZR 75-1 cells after 12 and 24 h of exposure to P.CNF.0, P.CNF.1 and P.CNF.5 scaffolds. Statistical significance: * *p* < 0.05, ** *p* < 0.01, *** *p* < 0.001, & *p* < 0.05, && *p* < 0.01.

To investigate the connection between 5-FU anti-tumor effects and inflammasome activity, we performed real-time PCR to analyze the changes in mRNA levels of p53 and caspase-1. Contact of MCF12A with P.CNF.5 resulted in an increased p53 mRNA profile, in comparison with P.CNF.0 (*p* < 0.01) (Figure 9). After 24 h, the same statistically significant upward profile of p53 mRNA was observed in contact with P.CNF.5, compared with the control (*p* < 0.05). A longer incubation period with P.CNF.0 scaffold led to an increase of p53 mRNA expression (*p* < 0.01), suggesting the interaction between 5-FU and p53 in normal breast cells.

Both breast cancer cell lines MDA-MB-231 and ZR 75-1 showed significantly increased p53 mRNA profiles in contact with P.CNF-enriched scaffolds, compared with the control cells. After 12 h, p53 mRNA expression in MDA/MB 231 cells put in contact with P.CNF.1 scaffold increased, in comparison with the simple P.CNF.0 scaffold (*p* < 0.05). Even higher expression of p53 mRNA was recorded in tumor cells contacted with P.CNF.5 (*p* < 0.001) due to the higher concentration of 5-FU embedded in the systems. After 24 h, the rise in gene expression was statistically significant in contact with both P.CNF.1 and P.CNF.5 scaffolds, when comparing with the expression registered in contact with P.CNF.0 scaffold (*p* < 0.01 and *p* < 0.001, respectively). Similarly, p53 mRNA levels were achieved by ZR 75-1 cells in contact with the proposed scaffolds. As observed, p53 mRNA expression increased in a time-dependent manner in contact with P.CNF.5 scaffold (*p* < 0.05).

**Figure 10:**
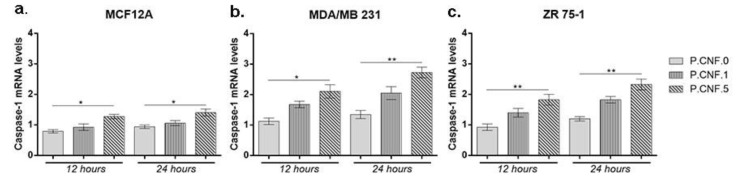
Caspase-1 profile of gene expression after 12 and 24 h of (**a**) MCF12A, (**b**) MDA/MB 231 and (**c**) ZR 75-1 cells’ contact with P.CNF.0-containing scaffolds. Statistical significance: * *p* < 0.05, ** *p* < 0.01.

The gene expression levels of inflammatory caspase-1 were determined in order to establish the activity of inflammasome complex after contact with 5-FU embedded in the scaffolds (Figure 10). For MCF12A cells, an overall profile showed higher caspase-1 mRNA levels in the presence of P.CNF.5, in comparison with P.CNF.0 (*p* < 0.05).

After 12 h, significant changes were observed at the level of caspase-1 expression for MDA/MB 231 cells that were put in contact with P.CNF.5 (*p* < 0.05). No statistically significant differences were registered between P.CNF.0 and P.CNF.1. This suggests that a higher 5-FU concentration is required for the inflammasome complex activation and, implicitly, for the maturation and release of caspase-1. Likewise, after 24 h of incubation there was a significant increase in the level of caspase-1 for P.CNF.5, as compared with the one registered for the simple scaffold (*p* < 0.01).

For the ZR 75-1 tumor cell line, significant differences were noted between P.CNF.0 and P.CNF.5 (*p* < 0.01) after 12 h and 24 h of incubation. The caspase-1 levels for P.CNF.5 were doubled, suggesting that 5-FU had a positive influence on the caspase-1 activity in higher concentrations. Slightly ascending—but statistically insignificant—caspase-1 expression profiles were registered for P.CNF.1, compared with P.CNF.0, suggesting that caspase-1 expression was induced in contact with P.CNF.1 as well, but at a much slower rate than on the P.CNF.5, due to the presence of a lower concentration of the drug.

### 3.9. Protein Expression Evaluation

**Figure 11:**
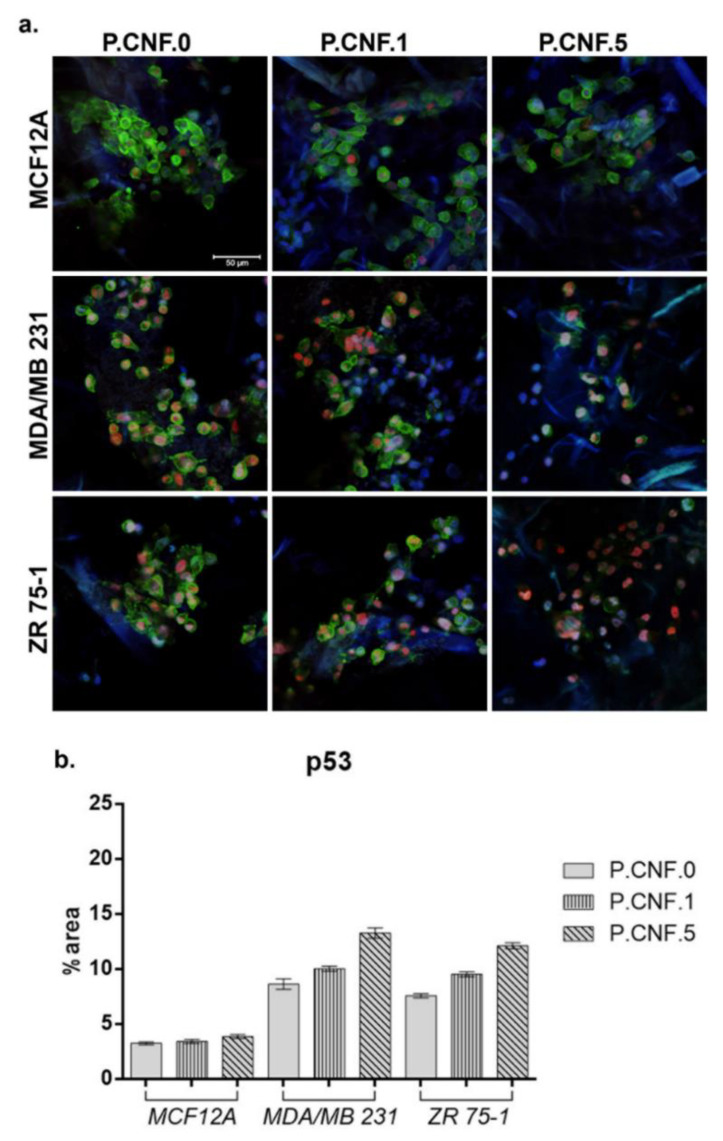
(**a**) p53 protein expression in MDA/MB 231 and ZR 75-1 cells, as compared to normal breast cells, MCF12A, all cultured in contact with P.CNF-based scaffolds. Scale bar 50μm. p53 is shown in red (p53-AF546), actin filaments are shown in green (phalloidin- FITC) and cell nuclei are shown in blue (Hoechst 33258). (**b**) Quantification of the p53 staining.

P53 and caspase-1 gene expression results were also confirmed at the protein level by confocal microscopy. After 24 h of contact with the proposed scaffolds, we evaluated the p53 protein level using fluorescence microscopy (Figure 11). On MCF12A normal breast cells, we observed the lowest concentration of 5-FU enhanced p53 expression, as compared with the P.CNF.0. In the cases of the MDA-MB-231 and ZR 75-1 cell lines, p53 was poorly expressed on the control, P.CNF.0, as compared with the expression on P.CNF.1 or P.CNF.5. Along with the increase of 5-FU concentration from 1 to 5 mg/mL, an increasing p53 expression profile was obtained, suggesting the anti-tumor effects of the drug on breast cancer cells. The highest levels of p53 were observed on the P.CNF.5 scaffold.

**Figure 12:**
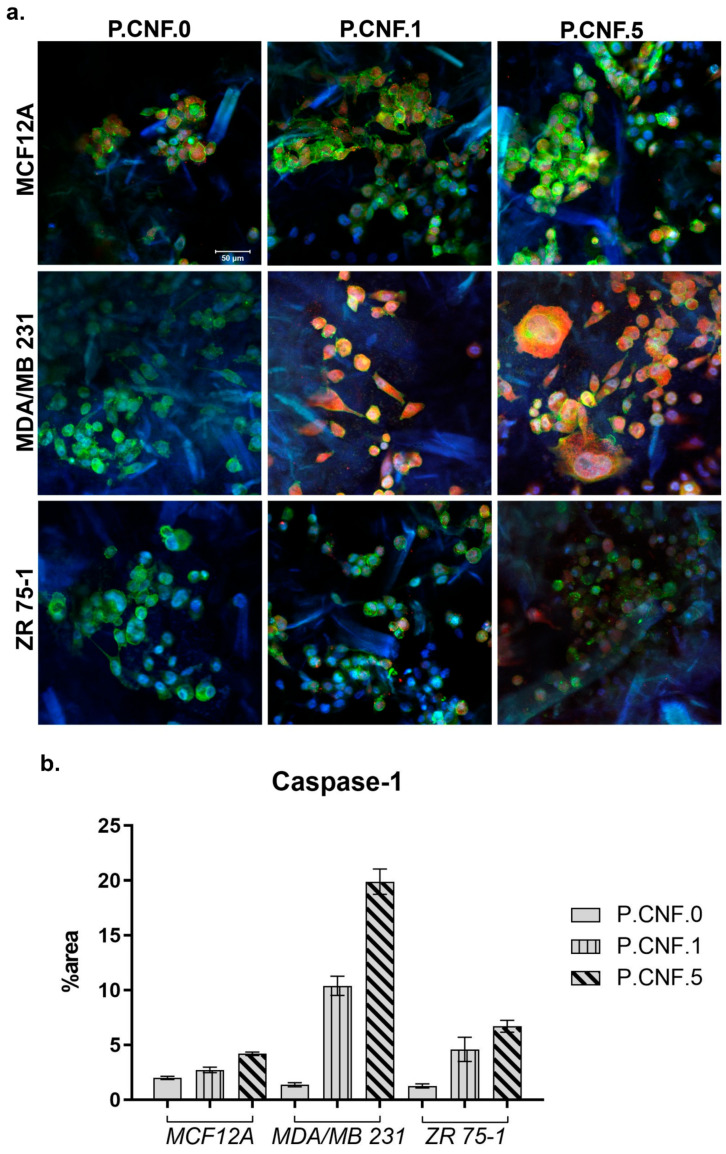
(**a**) Caspase-1 expression in MDA/MB 231 and ZR 75-1 breast cells, as compared to MCF12A normal breast cells, in contact with P.CNF-based scaffolds. Caspase-1 is shown in red (caspase-1-AF546), actin filaments are shown in green (phalloidin-FITC) and cell nuclei are shown in blue (Hoechst 33258). Scale bar 50 μm. (**b**) Quantification of caspase-1 staining.

To determine if 5-FU increased inflammasome complex activity, we examined the levels of active caspase-1 in breast cells after contact with P.CNF-enriched scaffolds (Figure 12). The results indicated that, in normal cells, caspase-1 activation was lower in P.CNF.1 and P.CNF.5, as compared with P.CNF.0. However, in MDA/MB 231 cancer cells, caspase-1 activation was significantly higher in P.CNF.1 and further increased in P.CNF.5. Based on the comparative levels of caspase-1 activity, we suggest that pyroptosis was initiated in MDA/MB 231 cells in contact with P.CNF.5. 

Additionally, 5-FU determine the caspase-1 activation in ZR 75-1 cells. The higher concentration of 5-FU embedded in P.CNF.5 further increased caspase-1 activity, compared with P.CNF.1. The obtained results support the idea that caspase-1 activation is a distinguished functional marker of pyroptosis.

**Figure 13:**
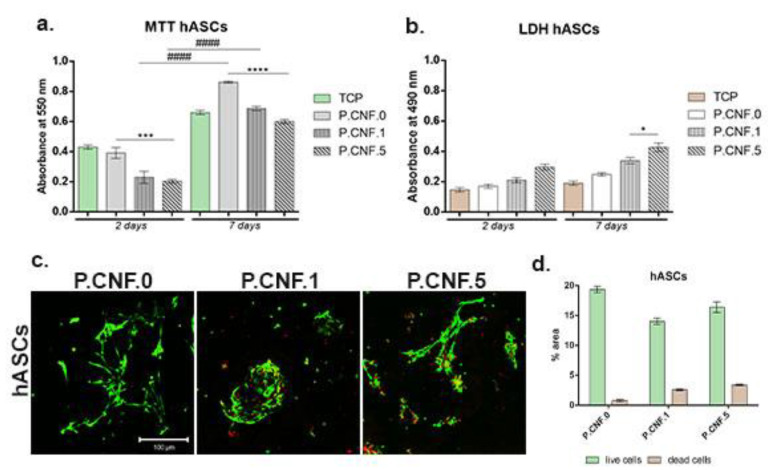
Biocompatibility evaluation performed for hASCs/P.CNF.0-containing scaffolds. (**a**) Cell viability profile obtained after 2 and 7 days of culture by MTT test. Statistical significance: *** *p* < 0.001, **** *p* < 0.0001, #### *p* < 0.0001. (**b**) Cytotoxicity levels exerted by scaffolds on hASCs after 2 and 7 days of culture. Statistical significance: * *p* < 0.05. (**c**) Qualitative Live/Dead staining showing live cells (green) and dead cell nuclei (red) of hASCs in contact with proposed scaffolds. Scale bar 100 μm. (**d**) Quantification of living cells (green) and dead cell nuclei (red) in all scaffolds.

Modern regenerative therapies use hASCs, due to their proliferation abilities and their high potential to differentiate into multiple cell lineages, including adipogenic lineage. Biocompatibility evaluation—performed for hASCs in contact with proposed biomaterials (Figure 13)—revealed that P.CNF.0-based scaffolds supported cell viability and proliferation rates. MTT profile revealed that, at 2 days after hASCs seeding on the control scaffold, P.CNF.0 presented a significantly higher viability (*p* < 0.001) than P.CNF.5. After 7 days of culture, a significantly higher (*p* < 0.0001) cell viability was registered on the tested P.CNF.0 control, as compared to P.CNF.5. Furthermore, the data suggested a growing proliferation pattern of hASCs from 2 to 7 days of culture in contact with these composites. We observed that cells exhibited significant (*p* <0.0001) proliferation during one week of culture—not only in contact with the controls, but also with the other composites.

The cytotoxic potential of the CNFs-based scaffolds was indicated by the levels of LDH released in the culture media. After 2 days of culture, low levels of toxicity were measured, which varied with the concentration of 5-FU added into the scaffolds. The cytotoxicity profile of the hASCs indicated that, during 7 days of cell culture, low LDH levels were measured in contact with the scaffolds. At 7 days post-seeding, a significantly high (*p* < 0.05) mortality rate was registered on P.CNF.5. Altogether, the proposed materials exhibited low cytotoxic potential due to the presence of the anti-tumor drug.

Fluorescent staining allowed for qualitative analysis of live and dead cells and cell distribution in contact with CNFs-based scaffolds. Live/Dead staining revealed that, after 7 days of culture, hASCs formed groups on P.CNF.0 scaffolds, which provided favorable conditions for hASC proliferation. On top of that, hASCs in contact with P.CNF.0 materials presented an elongated shape. Small amounts of dead cells were observed on hASCs/P.CNF.0 biosystems.

## 4. Discussion

Chronic inflammation at the local or systemic level and dysregulation of inflammasome activation can lead to the development of inflammatory diseases, neurodegeneration and cancer, implicitly in the progression and metastatic potential of BC. NLRP3 inflammasome activation, caspase-1 activation, release of IL-1β and IL-18 pro-inflammatory cytokines and gasdermin D-mediated pyroptotic cell death are important mechanisms involved in inflammation-induced cancer. The inflammasome and caspase-1 complex represents an active holoenzyme, as caspase-1 requires association with the inflammasome complex in order to maintain its activity [33]. Chen et al. studied the influence of inflammasome activation in breast tumor-associated microenvironments and *BRCA1* and *TP53* mutations. Their results indicated that inflammasome activation in *BRCA1* mutant tumor promoted tumor progression and that inhibition of inflammasomes could postpone tumor regression. Additionally, their study interpretation suggested the possibility of *BRCA1* mutant BC and metastasis inhibition through interference in inflammasome activation [34]. Detailed studies on in vivo models indicated that the loss of p53 in breast cancer cells determined WNT ligands’ secretion and IL-1β release, supporting an inflammatory reaction [35]. Moreover, IL-1β secretion stimulated breast cancer colonization as a result of WNT signaling pathway activation and the emergence of an inflammatory microenvironment [36].

Natural polymers have been used in various applications like wound healing, tissue regeneration, biomedical applications and drug delivery due to their low cost and their positive properties (biocompatibility, biodegradability and non-cytotoxicity). Considering all of these, natural polymers are capable of controlled drug release as a treatment method for cancer [37]. Several recent studies have focused on the encapsulation of drugs (5-FU, doxorubicin, docetaxel, paclitaxel, cisplatin etc.) into drug delivery systems for breast cancer treatment. The findings of those studies indicated that 5-FU loaded into polymers was effective at inhibiting MCF-7 breast cancer cells’ growth and proliferation, albeit in a concentration-dependent manner. Additionally, 5-FU and doxorubicin drug encapsulation displayed high cytotoxicity, suggesting the possibility of combinations of drugs in the treatment of multiple types of cancer, including BC [38]. Christensen et al. stated that TP53-mutated BC accumulated more 5-FU mutations than TP53 wild type BC [39]. Furthermore, recent work revealed that breast tumors that are deficient in the p53 DNA damage checkpoint regulatory pathway accumulated more 5-FU mutations [40]. However, in a study performed by Bertheau et al., it was suggested that TP53 mutation status may differ dependent on the chemotherapeutic treatment administered [41]. Besides, the relationship between TP53 mutation and chemotherapeutic treatment may be influenced by the types of drug, BC subtype, drug dose, treatment schedule and TP53 mutation detection method [42]. Geisler et al. affirmed that p53 was a key molecule in apoptosis initiation caused by the administration of antineoplastic drugs. In vivo studies revealed that 30% of BC patients presented TP53 mutation and p53 disturbances predicted therapy deficiency in BC [43].

In this study, we evaluated the potential of 5-FU-loaded ALG or PEC hydrogels as anti-tumor bioplatforms for biomedical applications. The biocompatibility of the CNFs was previously assessed and confirmed in a previous study [29]. The results of quantitative and qualitative viability tests indicated a strong cytotoxic effect of 5-FU on tumor cells, especially in 3D systems in which the concentration of 5 mg/mL 5-FU was added. Previous studies investigated the antineoplastic activity of 5-FU, its correlation with caspase-1 activation and the mechanism of inflammasome complex activation under the control of 5-FU treatment. The results suggested the capacity of 5-FU to limit tumor growth and recurrence, highlighting that the presence of anti-tumor drugs led to an increase in the number of ASC speck-positive cells, as compared to control cells. Additionally, the number of active caspase-1-positive cells increased after 12 h of exposure to 5-FU. The variable percentage of active caspase-1 cells induced by 5-FU was due to 5-FU metabolism, which involves a competition between elimination of the drug by dihydropyrimidine dehydrogenase and formation of active metabolites targeting the thymidylate synthase [44].

At the same time, the presence of 5-FU determined the increase of p53 and caspase-1 expressions at the gene and protein levels. The highest levels of caspase-1 were released in the culture medium that we detected in 3D systems composed of tumor cells and scaffolds enriched with 5-FU. Another study investigated the capacity of 5-FU to decrease cell viability and activate inflammasome complex in these cells. In vitro results suggested the kinetic activation of caspase-1 after 12 h of 5-FU administration. In vivo results registered the activation of caspase-1 after 24 h or 48 h. A plausible explanation is that chemotherapeutic agents require a conversion time into their metabolically active form [45]. Zhan et al. studied the capacity of a DNA tetrahedron to enhance 5-FU therapeutic efficacy and BC targeting. The obtained results suggested that tumor cell viability decreased in the presence of the DNA-based delivery system, as compared to normal breast cells. More detailed studies have indicated that the number of breast tumor cells in the S phase of the cell cycle were reduced after 24 h of treatment, as compared with the control. 5-FU anti-cancer drugs display pharmacodynamic activity and influence the expression of inflammatory caspases, which will further influence programmed cell death pathways [4].

In this study, ROS production was quantified by DCFDA (intracellular) and Ampliflu Red (extracellular H_2_O_2_) assays. The intracellular species detected included hydrogen peroxide, peroxyl, and hydroxyl radicals—and also, to a lesser degree, superoxide anions. Extracellular measurements of H_2_O_2_ comprised H_2_O_2_ produced outside of the cell by membrane-associated enzymes, for example NAD(P)H oxidases, and by H_2_O_2_ diffusing out of the cell. It is known that H_2_O_2_ can be transported out of the cell via channels in the cell membrane or via protein-mediated mechanisms [46].

The increased ROS production found in breast cells was mainly due to the 5-FU and was dependent on drug concentration. Even though the main anti-cancer mechanism of 5-FU is blocking DNA synthesis and replication, oxidative stress has also been proposed to play a key role. Previous studies have demonstrated that 5-FU increased intracellular levels of ROS in cancer cells and it is thought that oxidative stress may be responsible for many of the toxic effects of this drug [47]. It was previously reported that 5-FU induced excessive Ca^2+^ entry in MCF7 cells through activation of TRPV1, contributing to the rise of intracellular ROS production and depolarization of the mitochondria of intact cells and further inducing apoptosis through caspase-3 and 9 activations [48]. Pectin also contributed to ROS production in the tested breast cancer cells. Although pectins have antioxidant properties, they also exert anti-tumor activity [49].

Herein, we reported that pectin selectively generated ROS in MDA/MB 231 and ZR 75-1 human breast cancer cells, while no ROS production was detected in MCF12A normal cells. In accordance with our findings, recent studies have indicated that pectins are capable of inducing apoptosis in cancer cells without having any adverse effect on normal cells [42].

Another study reported that pectins can induce apoptosis in breast cells cancer cells via the collapse of the mitochondrial transmembrane potential, which functions up-stream of the caspase-dependent apoptosis and triggers cell arrest at the S and G1 or G2/M phases of the cell cycle, respectively [50]. Niture and Refai also indicated that pectin polymers induced apoptosis in various cancer cells by inhibiting MAP kinase activation, and via NF-kB inactivation [51].

This study’s results proved the anti-tumor efficacy of the tested materials, combining a drug (5-FU) with a pectin polymer and facilitating increased ROS generation in both intracellular and extra-cellular media. Moreover, our results suggested that components of the materials acted synergistically and selectively in breast cells, probably due to the pectin activity increasing the 5-FU toxicity in breast cancer cells.

This study demonstrated the cytotoxic effect of 5-FU on breast tumor cells, MDA/MB 231 and ZR 75-1 cell lines, as well as the consequences of 5-FU activity at the molecular level: increased expression of tumor suppressor p53 and activation of caspase-1, associated with an inflammatory profile.

## 5. Conclusions

In this study, we evaluated novel 3D hydrogels based on CNFs and pectin enriched with two 5-FU concentrations for their ability to suppress breast tumor cell growth and to promote pyroptosis of breast tumor cells as a result of inflammasome complex activation. The composites displayed porous morphologies, which facilitated normal breast cells, as well as hASCs’ viability and proliferation capacity. On the other hand, 5-FU’s presence in these composites enhanced extra- and intracellular ROS production by breast tumor cells, especially in MDA/MB 231 cells (triple negative subtype), as compared with ZR 75-1 cells (luminal subtype). The presence of an anti-tumor drug in the CNFs/5-FU hydrogels determined the activation of inflammasome in MDA/MB 231 and ZR 75-1, which promoted caspase-1 dependent cell death (pyroptosis). Additionally, 5-FU stimulated the expression of p53 and caspase-1 at the gene and protein levels. All of these findings confirmed the capacity of CNFs/5-FU hydrogels to modulate both breast tumor cells viability and inflammasome activity, contributing to developments in breast cancer therapy. In addition, the biocompatible properties of CNFs-based hydrogels and their interaction with hASCs recommend these 3D systems for future soft tissue engineering studies.

## Figures and Tables

**Table 1:** Composition of the CNFs-based hydrogels.

Polymer Blend Composition/100 mL			5-FU Concentration (mg/mL)	Sample Code
ALG (g)	PEC (g)	CNFs (g)		
3.39	-	1.13	0	A.CNF.0
3.39	-	1.13	1	A.CNF.1
3.39	-	1.13	5	A.CNF.5
-	3.39	1.13	0	P.CNF.0
-	3.39	1.13	1	P.CNF.1
-	3.39	1.13	5	P.CNF.5

## Data Availability

Not applicable.
