# Correction: Balahura et al. Cellulose Nanofiber-Based Hydrogels Embedding 5-FU Promote Pyroptosis Activation in Breast Cancer Cells and Support Human Adipose-Derived Stem Cell Proliferation, Opening New Perspectives for Breast Tissue Engineering. *Pharmaceutics* 2021, *13*, 1189

**DOI:** 10.3390/pharmaceutics17091122

**Published:** 2025-08-28

**Authors:** Liliana-Roxana Balahura, Sorina Dinescu, Mihaela Balaș, Alexandra Cernencu, Adriana Lungu, George Mihail Vlasceanu, Horia Iovu, Marieta Costache

**Affiliations:** 1 Department of Biochemistry and Molecular Biology, University of Bucharest, 050095 Bucharest, Romania; roxana.balahura@bio.unibuc.ro (L.-R.B.); mihaela.balas@bio.unibuc.ro (M.B.); marieta.costache@bio.unibuc.ro (M.C.); 2 Department of Immunology, National Institute for Research and Development in Biomedical Pathology and Biomedical Sciences “Victor Babes”, 050096 Bucharest, Romania; 3 Research Institute of University of Bucharest, 050107 Bucharest, Romania; 4 Advanced Polymer Materials Group, University Politehnica of Bucharest, 011061 Bucharest, Romania; alex.cernencu@gmail.com (A.C.); adriana.lungu@upb.ro (A.L.); vlasceanu.georgemihail@yahoo.ro (G.M.V.); horia.iovu@upb.ro (H.I.)

## Error in Figure

In the original publication [1], there was a mistake in Figure 12. An error occurred during the processing of the images and the construction of Figure 12 as two incorrect images were placed for ZR 75-1 cells cultured in contact with P.CNF.1 and P.CNF.5. The authors would like to replace the mistaken images with the correct ones corresponding to the ZR 75-1 cell line in Figure 12a and replace the corresponding columns showing the quantification of red fluorescence levels (caspase-1) in Figure 12b. The correct Figure 12 appears below. The authors state that the scientific conclusions are not affected by this change. This correction was approved by the Academic Editor. The original publication has also been updated.

## Figures and Tables

**Figure 12:**
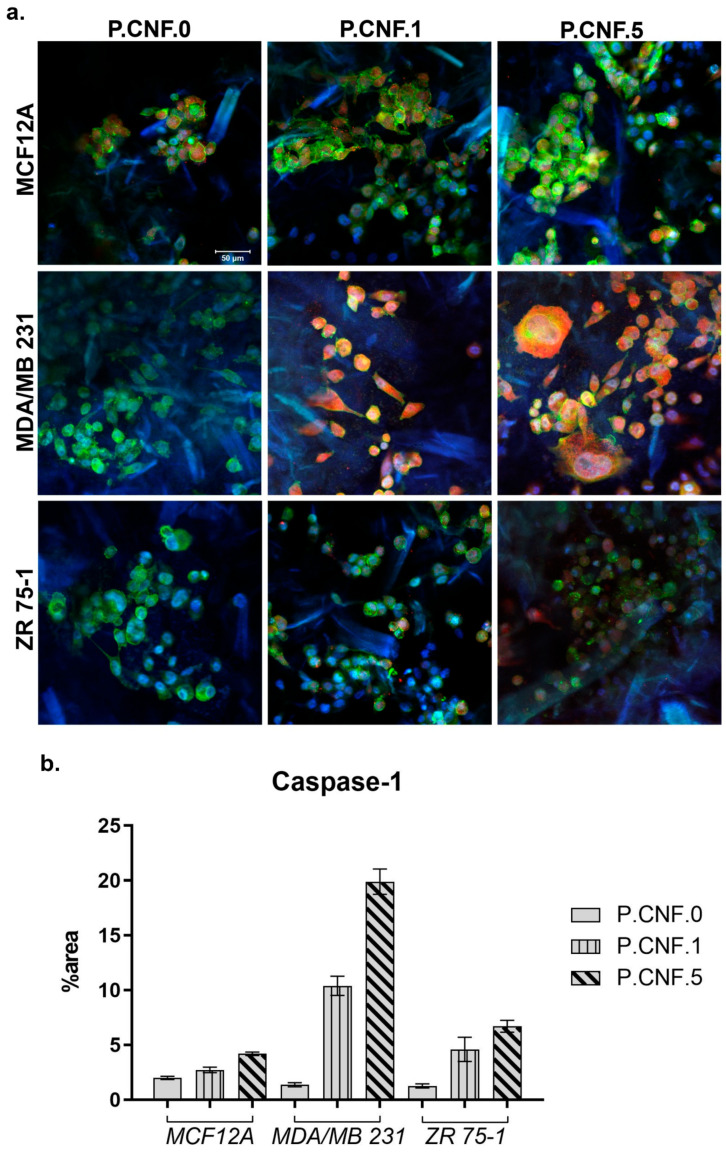
(**a**) Caspase-1 expression in MDA/MB 231 and ZR 75-1 breast cells, as compared to MCF12A normal breast cells, in contact with P.CNF-based scaffolds. Caspase-1 is shown in red (caspase-1-AF546), actin filaments are shown in green (phalloidin-FITC) and cell nuclei are shown in blue (Hoechst 33258). Scale bar 50 μm. (**b**) Quantification of caspase-1 staining.
